# Hepatectomy, RFA, and Other Liver Directed Therapies for Treatment of Breast Cancer Liver Metastasis: A Systematic Review

**DOI:** 10.3389/fonc.2021.643383

**Published:** 2021-03-26

**Authors:** Kevin Rivera, Dhiresh Rohan Jeyarajah, Kimberly Washington

**Affiliations:** School of Medicine, Texas Christian University/University of North Texas Health Sciences Center, Fort Worth, TX, United States

**Keywords:** breast cancer liver metastasis, hepatic resection, radiofrequency ablation, transarterial chemoembolization, transarterial radioembolization, liver directed therapies

## Abstract

**Background:**

The liver is the second most common site of breast cancer metastasis. Liver directed therapies including hepatic resection, radiofrequency ablation (RFA), transarterial chemo- and radioembolization (TACE/TARE), and hepatic arterial infusion (HAI) have been scarcely researched for breast cancer liver metastasis (BCLM). The purpose of this review is to present the known body of literature on these therapies for BCLM.

**Methods:**

A systematic review was performed with pre-specified search terms using PubMed, MEDLINE, EMBASE, and Cochrane Review resulting in 9,957 results. After review of abstracts and application of exclusion criteria, 51 studies were included in this review.

**Results:**

Hepatic resection afforded the longest median overall survival (mOS) and 5-year survival (45 mo, 41%) across 23 studies. RFA was presented in six studies with pooled mOS and 5-year survival of 38 mo and 11–33%. Disease burden and tumor size was lower amongst hepatic resection and RFA patients. TACE was presented in eight studies with pooled mOS and 1-year survival of 19.6 mo and 32–88.8%. TARE was presented in 10 studies with pooled mOS and 1-year survival of 11.5 mo and 34.5–86%. TACE and TARE populations were selected for chemo-resistant, unresectable disease. Hepatic arterial infusion was presented in five studies with pooled mOS of 11.3 months.

**Conclusion:**

Although further studies are necessary to delineate appropriate usage of liver directed therapies in BCLM, small studies suggest hepatic resection and RFA, in well selected patients, can result in prolonged survival. Longitudinal studies with larger cohorts are warranted to further investigate the effectiveness of each modality.

## Introduction

Among women worldwide, breast cancer is the most frequently diagnosed malignancy and second most common cause of cancer death (1). The liver is the primary site of spread in 15% of patients with metastatic breast cancer (2). Outcomes for breast cancer liver metastasis (BCLM) are grim with a median overall survival of 4–8 months, if untreated (2); 22 to 26 months following systemic chemotherapy alone with no reported 5-year survivors, and 37% 5-year survival after introduction of anti-HER2 therapy, although there is considerable variation of outcomes (3). Five-year survival rates for patients with BCLM with combination chemotherapy, immunotherapy, and hormone therapy are only 3.8–12% (median overall survival [mOS], 4–21 months) (4). Despite this, survival for patients with metastatic breast cancer has steadily improved over the last two decades (5) as evidenced by a single report of increased mOS from 17 months (1999) to 23.4 months (2008) (6).

BCLM management is an area of ongoing research. Advancement in liver directed therapies for the treatment of colorectal liver metastasis (CRLM) has open doors to the potential for improvement in survival for BCLM. The most direct intervention is hepatic resection, although the current oncologic dogma is that patients with BCLM are usually unresectable at diagnosis. Through successes with percutaneous and laparoscopic ablative technologies (radiofrequency and microwave) in CRLM, studies have emerged on its efficacy in treatment of BCLM. The introduction of trans-arterial chemoembolization (TACE) and radioembolization (TARE), the latter using yttrium-90 (Y-90), in the treatment unresectable liver metastasis has provided an option which focal therapies do not afford. Lastly, hepatic arterial infusion (HAI) has gained ground in CRLM. Although very few in number, this technique has been evaluated in management of BCLM.

The current body of literature on this topic is fragmented, with various small retrospective studies proving proof of concept for management of BCLM. There is no single published review examining the various methods of liver specific treatment options for BCLM. The purpose of this manuscript is to present the known body of literature on the topic of hepatic resection, RFA, TACE/TARE, and HAI for the treatment of BCLM. Using the available data, the authors will present an argument for use of multimodal therapies in highly selected cases of BCLM.

## Materials and Methods

### Registration, Search Strategy, and Study Selection

PROSPERO registration was obtained (CRD42020184009) for the systematic review after review of published works confirming lack of similar reviews in the literature.

Literature searches were performed by two independent reviewers (KW and KR) utilizing four electronic databases: PubMed, MEDLINE, EMBASE, and Cochrane Review from inception to May 2020. The following search terms were used: “liver metastasis,” “liver metastasis breast cancer,” “breast cancer liver metastases,” “breast cancer hepatic metastasis.” The outcome was a total of 9,957 results from the initial search.

Simple title review for the following key terms was performed: “hepatectomy,” “hepatic resection,” “Radiofrequency Ablation,” “Microwave Ablation,” “Transarterial chemoembolization,” “Transarterial radioembolization,” and “Hepatic Arterial Infusion.” All duplicates were removed resulting in a total of 195 studies.

### Inclusion and Exclusion Criteria

The inclusion criteria were as follows: (1) contains study of interventions in the treatment of BCLM; (2) publication as a full research article in English language, (3) related surgery/procedure detail and outcome indicators were reported.

**Figure 1:**
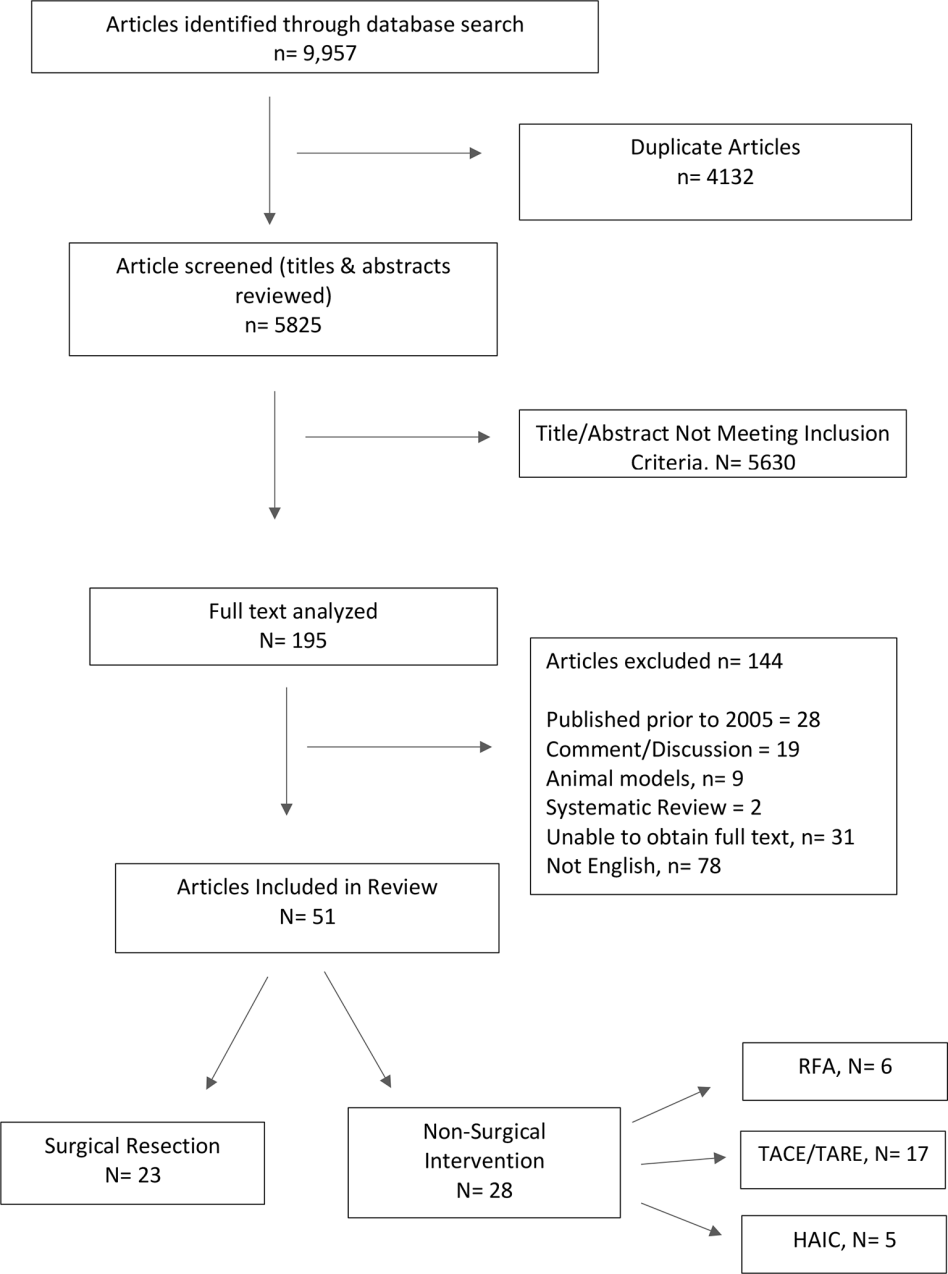
PRISMA Flow Diagram.

One hundred thirty-nine studies were excluded (**Figure 1**). After exclusions, 51 studies were chosen for inclusion.

### Data Extraction and Management

Fifty-one studies were reviewed in full text format. Data was extracted directly from the text of the article and collated into the following categories: study design, type of intervention, total patients, mean age, histology of primary tumor, hormone receptor status of primary tumor, adjuvant therapy, extent of hepatic resection (major *vs* minor), resection margin status, time from primary to liver metastasis, RECIST response, post-procedure complications, 30-day mortality, median overall survival, disease free survival, 1-, 3-, and 5-year survival. All relevant text, tables, and figures were reviewed for data extraction.

For safety and effectiveness outcomes, overall rates of each complication were calculated using the unweighted median figures given in each study. In studies where a mean was presented when a median value is most commonly used, the data will be provided for table completion, but notation is made to identify the difference in reporting method. Consensus discussion resolved all discrepancies between reviewers.

### Quality and Methodological Assessment

There were no randomized studies found in the literature search. The non-randomized studies comparing two interventions were evaluated using the Risk of Bias in Non-randomized Studies–of Interventions (ROBINS-I) Assessment Tool (7). This tool was utilized to examine and measure seven specific bias domains: confounding, selection of participants, classification of interventions, deviation from intended interventions, missing data, measurement of outcomes, and selection of reported result. There were a total of five studies that met criteria: hepatic resection *versus* systemic chemotherapy (8, 9), laparoscopic RFA *versus* systemic chemotherapy (10), and TACE *versus* TARE (11), TACE *versus* systemic chemotherapy (12) (**Table 1**). All other included studies were identified as retrospective or, rarely, prospective and observational, and therefore offered no comparison of interventions. These studies are subject to the biases inherent to their study design.

**Table 1:** ROBINS-I Assessment of Non-randomized studies: D1, Bias due to confounding; D2, Bias in selection of participants into the study; D3, Bias in classification of interventions; D4, Bias due to deviations from intended interventions; D5, Bias due to missing data; D6, Dias in measurement of outcomes; D7, Bias in selection of the reported result; X, high; –, some concern; +, low.

	D1	D2	D3	D4	D5	D6	D7	Overall
Mariani et al. (8)	–	–	+	+	+	+	+	+
Ruiz et al. (9)	X	X	+	+	+	+	–	–
Tasci et al. (10)	–	–	–	+	+	+	+	–
Chang et al. (11)	X	–	+	+	+	+	+	–
Duan et al. (12)	–	–	–	+	+	–	+	–

### Statistical Analysis

All studies were case series comprised of retrospective studies (n = 45) and prospective studies (n = 7). The outcomes measures varied widely, precluding meta-analysis. Therefore, the results of this review are presented in descriptive terms only.

## Results

### Hepatic Resection

Hepatic resection is the most published intervention for management of BCLM (**Table 2**). Of 23 studies, 13 provided mean diameter of liver metastasis (8, 9, 14, 19, 21, 24–26, 29–32). The median tumor size was 3 cm (1.8–5.2 cm). Twenty-one studies provided detail of surgical resection (8, 9, 13, 16–32)—there were a total of 437 major hepatectomies (47.1%). Approximately 597 (79.6%) obtained R0 resection (8, 9, 16–21, 23, 25, 27–30, 32). Mortality rates were available for 19 studies (8, 9, 13–21, 23, 25, 27–32) which was consistently low across with a median of 0% (0–5.5%). It is worth noting, however, that these values are difficult to compare since mortality rates were not standardized. Some reported “perioperative” mortality, while others reported “in-hospital” mortality, or “30-day” mortality. Tumor histology was provided in 12 studies, totaling 424 patients with ductal carcinoma, 77 with lobular carcinoma, and 10 mixed or other histology (8, 15–17, 19–25, 28, 30, 32). No studies stratified outcomes based on primary tumor histology. Thirteen studies provided detail on hormone status of the primary tumor (8, 9, 14, 17, 20–25, 28, 30–32). Hormone receptor positive tumors totaled 431 in the included studies. Her2 positive tumors totaled 117 patients. Nineteen studies reported mean time between diagnosis of primary of liver metastasis (8, 9, 13, 15–17, 19–32). Six studies provided details of receptor status at time of hepatectomy (16, 17, 20, 25, 28, 30). One hundred eighty tumors were estrogen receptor positive, 125 tumors progesterone receptor positive, and 54 tumors Her2 positive. The median time to diagnosis of liver metastasis was 41.7 months. Twenty-two studies provided a mOS after hepatectomy (8, 9, 13–28, 30–32). The mOS was 45 months (range 19–134.5 months). Five-year survival data was provided for 22 studies (8, 9, 13–19, 21–32). Median 5-year survival was 41% (21–78%). Twenty studies reported univariate and multivariate analysis in an attempt to ascertain factors that affect overall survival of their cohorts (17, 18, 20, 21, 23–28, 30–32). Factors identified on univariate analysis that improved overall survival include: partial response to neoadjuvant chemotherapy, hormone receptor positivity, R0 resection, disease-free interval >2 years, HER2 positive primary tumor treated with trastuzumab, age >49.1 years at diagnosis of liver metastasis, age >45.2 years at mastectomy, Pringle maneuver during hepatectomy, size of liver metastasis <4 cm, negative portal lymph nodes, solitary metastasis, and uni-lobar metastasis distribution.

**Table 2:** Hepatic Resection for BCLM.

Study	Year, Location	Study Design	# of Patients	Mean Tumor Size, cm	Major Hepatectomy	Minor Hepatectomy	R0 Margin	Mean Disease Free (Prior to Hepatectomy), mo	Mean Disease Free (Post Hepatectomy), mo	Overall Survival, mo	5-Year Survival, %
Sakamoto et al. (13)	2005, Japan	Retrospective	34	2.5	15	19	–	22.8	–	36	21
Adam et al. (14)	2006, France	Retrospective	85	2.8	54	31	–	34	12	38	41
Adam et al. (15)	2006, France	Retrospective	460	–	–	–	–	–	–	45	41
Caralt et al. (16)	2008, Spain	Prospective	12	–	5	7	9	54.6	29.8	41.3	33
Lubrano et al. (17)	2008, France	Retrospective	16	–	9	7	14	54	–	42	33
Thelen et al. (18)	2008, Germany	Retrospective	39	–	20	34	28	–	–	74	42
Hoffmann et al. (19)	2010, Germany	Retrospective	40	3	22	19	32	40.8	8.6	58	48
Abbott et al. (20)	2012, USA	Retrospective	86	–	53	33	77	14.2	14.2	57	–
Van Walsum et al. (21)	2012, Netherlands	Retrospective	32	2.5	13	19	29	33	11	55	37
Kim et al.* (22)	2013, S. Korea	Retrospective	13	–	1	12	–	62.5	–	25.2	49.2**
Kostov et al.* (23)	2013, Bulgaria	Retrospective	42	–	29	13	35	–	29.4	43	38.5
Mariani et al. (8)	2013, France	Retrospective	51	1.8	14	37	44	34	–	91^	50.1
Zegarac et al. (24)	2013, Serbia	Retrospective	32	2.8	17	13	–	25	22.5	37	34.4
Dittmar et al. (25)	2013, Germany	Retrospective	34	4	23	5	21	33.5	–	36	26
Treska et al. (26)	2014, Cz Republic	Retrospective	13	5.2	4	9	–	48	–	26.4	11.4
Weinrich et al. (27)	2014, Germany	Retrospective	21	–	6	15	18	55	–	53	33
Ruiz et al. (9)	2015, France	Retrospective	120	3.4	57	63	63	33.45	–	19	47
Vertriest et al. (28)	2015, Belgium	Retrospective	27	–	3	24	24	62	47	116	78
Kobryn et al. (29)	2016, Poland	Retrospective	11	4.1	2	8	10	42	–	–	9.1
Margonis et al. (30)	2016, USA	Retrospective	131	3	43	73	108	34	24	53.4	75.2**
Cheung et al. (31)	2019, China	Retrospective	21	2.1	15	6	21	41.7	13.7	134.5	58.9
He et al. (32)	2020, China	Retrospective	67	4.2	32	35	64	51.21	13.47	57.59	32.2

Twenty-three published studies since 2005 presenting survival data after hepatic resection. *Includes 2 patients with combined RFA + resection; **3-year survival. ^Reported median survival, not medial overall survival.

### Radiofrequency Ablation (RFA)

Percutaneous and laparoscopic RFA (perc-RFA and lap-RFA) for the treatment of BCLM was presented in six studies (**Table 3**). All studies included patients who received primary treatment with systemic chemotherapy, targeted therapies, or immunotherapy. Five studies presented results of perc-RFA (33–37) and one study of solely lap-RFA (10). There were a total of 186 patients in the pooled cohort. Three studies provided histology data on the primary tumor (10, 35, 37). There were 92 ductal carcinoma, 7 lobular carcinoma, and 27 mixed or other histology. Fifty-one tumors were hormone receptor positive and 32 tumors were Her2 positive. The mean tumor size was 2.9 cm (1.9–3.7 cm) as published in five studies (10, 34–37). Five studies provided median interval between diagnosis of primary and liver metastasis (10, 33, 34, 36, 37). The median interval was 47.6 months (22–87 months). Five studies provided mOS (10, 33, 35–37), which was 38 months (26–60 months). Four studies provided 5-year survival ranging from 11 to 33% (10, 33, 35, 37).

**Table 3:** Radiofrequency Ablation.

Study	Year, Location	Study Design	Method	# of Patients	Mean Tumor Size, cm	Interval From 1° and Liver Disease, mo	Mean Progression Free Survival, mo	Median Overall Survival, mo	5-Year Survival, %	Included Pts With EHD*
Sofocleous et al. (33)	USA, 2007	Retrospective	Percutaneous	12		83		60	33	y
Gunabushanam et al. (34)	India, 2007	Retrospective	Percutaneous	14	1.9	22		NR^	–	Y
Meloni et al. (35)	Italy, 2009	Prospective	Percutaneous	52	2.5	–		29.9	32	Y
Carrafiello et al. (36)	Italy, 2011	Retrospective	Percutaneous	13	3.5	87		38	–	Y
Tasci et al. (10)	USA, 2013	Retrospective	Laparoscopic	26	3.7	26.5		48	29	Y
Bai et al. (37)	China, 2018	Retrospective	Percutaneous	69	2.9	47.6	24	26	11	Unknown

*EHD defined stable disease. ^NR, not reported.

### Transarterial Chemoembolization (TACE) and Transarterial Radioembolization (TARE)

TACE has been described in eight studies, including one comparing TACE to TARE (**Table 4**). All studies provided RECIST response data (362 patients). There were an astonishing 26 complete responders (7.2%); 132 partial responders (38.1%); 133 patients (36.7%) with stable disease; and 58 patients (16%) had disease progression. mOS data from six studies (11, 48–52) showed a median of 19.6 months (4.6–32 months). One-year survival data was provided in four studies (12, 47, 49, 51) ranging from 32 to 88.8%. Cox regression analysis was performed in two studies (12, 47) revealing the following characteristics suggestive of improved outcomes on univariate analysis: N0 status, stage I or II disease, and Child-Pugh A at diagnosis of liver metastasis.

**Table 4:** Transarterial Chemoembolization (TACE) and Transarterial Radioembolization (TARE).

Study	Year, Location	Study Design	# of Patients	TACE/TARE	Regimen	Complete Response	Partial Response	Stable Disease	Progressive Disease	Median OS, mo
Bangash et al. (38)	2007, USA	Prospective	27	TARE	90Y	–	9	12	2	–
Coldwell et al. (39)	2007, USA	Retrospective	44	TARE	90Y	0	23	8	2	–
Jakobs et al. (40)	2008, USA	Retrospective	23	TARE	90Y	0	14	8	1	11.7
Fendler et al. (41)	2012, Germany	Retrospective	58	TARE	90Y	–	–	–	–	10.3
Haug et al. (42).	2012, Germany	Retrospective	58	TARE	90Y	–	11	27	5	11.8
Cianni et al. (43)	2012, Italy	Retrospective	52	TARE	90Y	0	29	18	5	11.5
Gordon et al. (44)	2014, USA	Retrospective	75	TARE	90Y	–	24	43	1	6.6
Saxena et al. (45)	2014, Australia	Retrospective	40	TARE	90Y	2	10	15	11	13.6
Chang et al. (11)	2018, USA	Retrospective	30	TARE	90Y	0	12	2	15	12.9
Deipolyi et al. (46)	2018, USA	Retrospective	31	TARE	90Y	7	11	6	2	10.9
Li et al. (47)	2005, China	Retrospective	28	TACE	Fludrouracil; 5-FUDR, cisplatin	2	8	13	5	–
Cho et al. (48)	2010, USA	Retrospective	10	TACE	Adriamycin, Cisplatin; Gemcitabine, oxaliplatin		2	2	5	26
Duan et al. (12)	2011, China	Retrospective	44	TACE	Iodized oil/doxorubicin; gelatin sponge particles	14	12	11	7	–
Vogl et al. (49)	2011, Germany	Prospective	161	TACE	Mitomycin C, gemcitabine; mitomycin C alone	–	92	69	–	32.5
Martin et al. (50)	2012, USA	Retrospective	40	TACE	Doxorubicin	9	11	12	6	47
Eichler et al. (51)	2013, Germany	Prospective	43	TACE	Gemcitabine	–	3	16	22	10.2
Gruber-Rouh et al. (52)	2017, Germany	Retrospective	19	TACE	Gemcitabine, mitomycin C	–	7	9	3	13.2
Chang et al. (11)	2018, USA	Retrospective	17	TACE	Doxorubicin	1	3	1	10	4.6

TARE was presented 10 published studies (**Table 4**). Y-90 is the most commonly used radioembolizing product. A total of nine studies provided RECIST response data (11, 38–40, 42–46). There were nine complete responders (2.3%) out of the 380 total patients. One hundred forty-three patients (37.6%) had partial response; 139 patients (36.5%) had stable disease; and 44 patients (11.5%) had disease progression. Eight studies provided a mOS (11, 40–46). The mOS of the pooled data was 11.5 months (6.6–13.6 months). Three studies provided 1-year survival data (39, 44, 45) which ranged from 34.5 to 86%. COX regression analysis was reported in four studies (11, 42, 45, 46). Through univariate analysis, SUVmax response, lower volume of hepatic parenchyma involvement, chemotherapy after TARE, and radiologic response to treatment were identified as factors improving overall survival. A single study identified presence of PI3K mutation as a factor increasing the likelihood of radiologic response after treatment.

### Hepatic Arterial Infusion (HAI)

Five studies were identified in which HAI was used for the treatment of BCLM (**Table 5**). Four studies reported RECIST response rates. There were two patients (1.2%) in the pooled cohort (N = 158) with complete response; 48 patients (30.4%) with partial response; 49 patients (31%) with stable disease; and 60 patients (38%) with progressive disease. Two studies presented univariate and multivariate analysis data (56, 57). The findings on univariate analysis suggest the following factors contribute to overall survival: ECOG status, hormone receptor status, maximum size of liver metastasis, and response to systemic chemotherapy. The median PFS of the pooled data was 5.45 months (2–8.4 months). mOS data was presented in all studies (53–57). The mOS for all pooled data was 11.3 months (7–19.3 months).

**Table 5:** Hepatic Arterial Infusion (HAI).

Study	Year, Location	Study Design	# of Patients	Objective Response Rate, %	Median OS, mo	Median PFS, mo	EHD	Chemotherapy Regimen
Maes et al. (53)	2008, Belgium	Retrospective	30	33.3	7.3	3	19	MMC
Nielson et al. (54)	2012, Denmark	Prospective	16	50	19.2	7.9	14	Oxaliplatin
Tewes et al. (55)	2017, Germany	Retrospective	70	20	7	2	62	Mitomycin and melphalane
Hsaio et al. (56)	2018, Taiwan	Retrospective	42	47	19.3	8.4	–	Mitoxantrone, folinic acid, 50FU, and cisplatinum
Furuta et al. (57)	2020, Japan	Retrospective	57	63	11.3	–	–	5-fu, epirubicin, and MMC

Demonstrates various chemotherapy regimen.

## Discussion

### Early Detection, Predictive Models, and Treatment of BCLM

Hepatic resection is a rare option for BCLM as the vast majority of patients have bilobar, unresectable disease at diagnosis. This difference in discovery of liver metastasis at a resectable state between CRLM and BCLM could stem from the lack of surveillance recommendations for BCLM. National Comprehensive Cancer Network (NCCN) guidelines recommend follow-up computed tomography (CT) of the abdomen and pelvis every 6–12 months for 5 years in stage II and higher colon cancer. Ninety-three percent of all CRLM were diagnosed within 3 years of the primary (58). Breast cancer, on the other hand, carries no recommendation for imaging surveillance for liver metastasis. Median time to liver metastasis from all included studies in this review is 41.35 months (22–83 months), comparable to that of CRLM.

As there are many patients with metastatic breast cancer without liver disease, there must be tumor specific factors that promote liver metastasis. Kimbung, et al. identified 17 liver metastasis-selective genes of prognostic relevance in early breast cancer which independently identify patients at higher risk within both luminal A and luminal B molecular subtype (59). This group also identified the novel role for claudin-2 as a prognostic biomarker for the likelihood of breast cancer recurrence, specifically liver recurrence (60). Lin et al. developed a nomogram for prediction of liver metastasis in breast cancer patients based on eight characteristics at diagnosis of primary disease: sex, histology, nodal involvement, histologic grade, ER/PR/Her2 status, and age at diagnosis (4). There has been no prospective external validation of this nomogram found within the literature.

This presents a major area of further research, as developing a reliable predictive model for development of liver metastasis based on characteristics at diagnosis of primary disease could pave the way for selective surveillance for liver metastatic disease. This would, in theory, result in earlier diagnosis of BCLM, which could, in turn, broader treatment options and potential survival advantage.

### Hepatic Resection

Hepatic resection is the mainstay in definitive treatment for primary liver malignancies and metastatic disease to the liver. Although few metastatic diseases afford an aggressive approach, hepatic resection is gaining more traction, most notably in CRLM.

The longest mOS after hepatic resection is reported as 134.5 months as reported by Cheung et al. (31). Although a small study (21 patients), all underwent R0 resection. The cause for prolonged survival in this cohort compared to others is likely due to patient selection. The patient population was younger, with median age of 45 at diagnosis and selected for only those with oligometastatic liver disease. On univariate analysis, estrogen receptor positivity was found to be a protective factor for overall survival (OR = 0.159; 95% CI: 0.030–0.848; P = 0.067). Alternatively, triple negative status had a negative impact on overall survival (OR = 5.580; 95% CI: 1.210–25.731; P = 0.027). These findings are similar to those reported by Vertriest et al. (28), including a high R0 resection rate (88.9%), long disease-free interval prior to liver metastasis (62 months), long mOS after hepatectomy (116 months), and high 5-year survival (78%). Similar to that employed in the Cheung study, only those without macroscopic EHD, with disease-free interval >12 months, no more than three liver lesions, and response to systemic chemo- or hormone therapy were offered resection.

Of note, Sadot et al. published their experience with a combination of hepatic resection and ablation for management of BCLM (61). The retrospectively reviewed 167 patients with isolated BCLM comparing outcomes with those who received standard chemotherapy alone. There were 69 patients who underwent resection [42], percutaneous ablation [29], combination resection and ablation [2], and 98 patients who received standard chemotherapy. The hepatic tumor burden was less and the time from primary diagnosis to resection was longer in the surgical intervention group. The surgical group had a median recurrence free period 28.5 months and 10 patients were recurrence free at 5 years. They found no difference in mOS or 5-year survival between the medical and surgical cohorts. The obvious limitation of this study is its retrospective nature. Additionally, it is worth mentioning that the groups were significantly dissimilar in the most important ways. As mentioned by the authors, the tumor burden was lower in hepatic resection patients, but more importantly, the percutaneous ablation group were, by nature, not candidates for resection, whether due to location of metastasis or comorbidities. As this published study is an outlier, it begs further research to evaluate the role of hepatic resection and RFA in the treatment of this disease. Further studies are needed to determine if patients who are deemed resectable but go on to systemic chemotherapy only have similar survival to those who are resected.

Based on the evidence presented, it appears that younger patients with resectable tumors less than 3 cm in size, good functional status, long interval between diagnosis of the primary and liver metastasis, and no extra-hepatic disease (or stable bone-only EHD) have significantly improved survival after hepatic resection. Further studies are necessary to clearly delineate which subset of patients gain the greatest survival benefit from hepatic resection.

### Radiofrequency Ablation (RFA)

RFA was used in combination with systemic chemotherapy, metastasectomy, hormonal therapy, or radioembolization. The largest study to date was published by Bai et al. study (37), which involved 69 patients over a 15-year period. The mOS from diagnosis was 36 months. Approximately half the cohort had up to four liver metastases. Additionally, approximately half the cohort had extrahepatic disease at time of RFA. Due to these factors, the mOS was expected to be lower than other studies retrieved. Nevertheless, the 5-year survival from time of diagnosis was 20.7%, which gives cause for further research.

Tasci et al. is the sole study reviewing their experience with lap-RFA (10). There were no reported complications. Furthermore, mOS and 5-year actuarial survival compared with standard chemotherapy alone were 48 months *vs* 9 months and 29 *vs* 0%, respectively. The mOS, 5-year actuarial survival, and clinical benefits of this treatment modality over percutaneous RFA seem to favor lap-RFA. Yet, there is a need for a study that directly compares lap-RFA and perc-RFA to determine if there is a statistically significant difference in outcomes.

As RFA is often used in the management of other malignant liver diseases when hepatic resection is not feasible and the burden of disease is small, the utility of this modality may follow similar guidelines in BCLM. Further longitudinal studies among larger patient populations are warranted to characterize the role of RFA as an adjunct treatment for a subset of patients with BCLM.

### TACE/TARE

Within the past 15 years, several studies have confirmed the efficacy of TACE and TARE in the treatment of BCLM. Although few in number, these studies demonstrate similar or improved survival compared to systemic chemotherapy alone.

In published BCLM studies, TACE rarely resulted in complete radiographic response. Most notably, a study by Duan et al. (12). showed their center’s experience with 44 patients receiving TACE with doxorubicin. TACE resulted in 14 patients with complete radiologic response. This is an astonishing number of complete responders. However, there was no mOS or progression free survival data, which could be of value for those in the complete response group. The overall 1- and 3-year survival was 76.2 and 47.6% respectively, significantly higher than their control group (48.1 and 7.4%). From this body of data in the setting of unresectable BCLM, it is clear that the role of TACE in the treatment algorithm cannot be understated, however, more research is necessary to delineate which patients would most benefit.

Similar to TACE, TARE rarely produces complete radiographic response. Usage of Y-90 resin or glass microspheres allows for high dose radiation targeted to a specific area without radiation effects to the surrounding organs. Saxena et al. reported their experience with TARE in unresectable, chemo-resistant BCLM (45). The cohort consisted of 40 patients, of which six underwent hepatic resection prior to TARE. The median time to progression was 6.8 months, mOS was 13.6 months, and 1- and 2-year survival of 61 and 39%. This cohort underscores the impact TARE could make on overall survival in patients with chemo-resistant disease. Nevertheless, further studies are necessary to determine true survival data for patients with chemo-resistant disease.

The most obvious difficulty with interpreting this data is that for TACE, no two chemoembolization regimen were the same, precluding our ability to combine the data in a reproducible fashion. It appears, however, that there is some benefit of these therapies in patients with good ECOG status and unresectable, chemo-resistant disease. This is an area where further studies are necessary, in light of the fact that there are a considerable number of patients with complete and/or partial response.

### Hepatic Arterial Infusion

Similar to the previously discussed methods, HAI for the treatment of BCLM remains in its infancy. The earliest study reviewing HAI for BCLM by Arai et al. was published in 1994 with 56 patients (62). The chemotherapy regimen consisted of 5-FU, Adriamycin, and mitomycin C. Complete response was observed in 11 patients (19%), partial response in 33 (58.9%), stable disease in 6 (10.7%), and progressive disease in 4 (7.1%).

HAI and TACE share similar challenges, most notably determining appropriate chemotherapy regimen. The most intensive therapy regimen was reported by Furuta et al., which included 5-FU, epirubicin, and mitomycin C (57). This study also had the most robust objective response rate. Further research is required to determine the optimal regimen for treatment of BCLM, however it appears HAI could be of value in the management of unresectable BCLM as either an adjunct therapy or to convert to resectability.

### Limitations

As stated previously, there were no randomized controlled trials found in the literature on the topic of hepatic resection, RFA, TACE/TARE, or HAI for the treatment of BCLM. Therefore, all provided studies are retrospective in nature, placing considerable risk of bias in the presented studies. Selection bias is of great concern across the published studies as patients undergoing hepatic resection, for example, were highly specific: often those without extrahepatic disease (an uncommon phenomenon) or those with stable extrahepatic bone-only disease. Additionally, there is publication bias, as there was only one study published which suggested non-superiority of hepatic resection and RFA compared with chemotherapy alone (61). This may be partially related to the current dogma in treatment of BCLM, and therefore, treatment is largely systemic chemotherapy alone. Also, it cannot be ignored that this may be because others have attempted hepatic resection, RFA, TACE/TARE, and HAI with poor results which were not published. Lastly, within the literature provided, there can be lead time bias based on the surveillance methods employed by various oncology departments. This could result in the semblance of prolonged survival after intervention, when none exists if a standardized surveillance method was used. In light of the various limitations of these studies, further controlled trials and well-designed studies are necessary to answer this very important question. Despite the biases presented, however, some broad conclusions can be made in regards to which patients and under which circumstances each of these treatment methods could be studied further.

## Conclusion

In conclusion, interventional management of BCLM is in the earliest stages of development, particularly in determining the indications for hepatic resection, RFA, TACE/TARE, and HAI. Several studies confirm the safety and efficacy of each intervention, however there are many questions that require further investigation to determine the appropriate usage of each intervention as well as optimal chemotherapeutics for TACE and HAI. Hepatic resection may improve survival in highly selected patients with BCLM who are younger at diagnosis, have smaller tumor size, and no extra-hepatic disease. RFA requires further study, both laparoscopically and percutaneously for this disease, as it is unclear the benefit of this intervention either in combination with hepatic resection or alone. TACE may improve survival in patients with unresectable, chemo-resistant BCLM, however, there is no consistent chemoembolization regimen. TARE, on the other hand, does not show improved survival consistently across published studies. Lastly, HAI requires further research to determine appropriate selection for usage and chemotherapy regimen which is varied in the literature. This is a vast area of further research and the authors hope this review will encourage further study on these topics.

## Author Contributions

All authors listed have made a substantial, direct, and intellectual contribution to the work and approved it for publication.

## Conflict of Interest

The authors declare that the research was conducted in the absence of any commercial or financial relationships that could be construed as a potential conflict of interest.
